# Synthesis and Crystal Chemistry of Octahedral Rhodium(III) Chloroamines †

**DOI:** 10.3390/molecules25040768

**Published:** 2020-02-11

**Authors:** Kirill V. Yusenko, Aleksandr S. Sukhikh, Werner Kraus, Sergey A. Gromilov

**Affiliations:** 1 BAM Federal Institute for Materials Research and Testing, Richard-Willstätter Str. 11, D-12489 Berlin, Germany; w.kraus@bam.de; 2 Nikolaev Institute of Inorganic Chemistry, Lavrentiev ave. 3, 630090 Novosibirsk, Russia; a_sukhikh@niic.nsc.ru (A.S.S.); grom@niic.nsc.ru (S.A.G.); 3 Novosibirsk State University, Pirogova str. 2, 630090 Novosibirsk, Russia

**Keywords:** rhodium complexes, ligand substitution, crystal structure, pseudo-translational sublattices

## Abstract

Rhodium(III) octahedral complexes with amine and chloride ligands are the most common starting compounds for preparing catalytically active rhodium(I) and rhodium(III) species. Despite intensive study during the last 100 years, synthesis and crystal structures of rhodium(III) complexes were described only briefly. Some [RhCl*_x_*(NH_3_)_6-*x*_] compounds are still unknown. In this study, available information about synthetic protocols and the crystal structures of possible [RhCl*_x_*(NH_3_)_6−*x*_] octahedral species are summarized and critically analyzed. Unknown crystal structures of (NH_4_)_2_[Rh(NH_3_)Cl_5_], *trans*–[Rh(NH_3_)_4_Cl_2_]Cl⋅H_2_O, and *cis*–[Rh(NH_3_)_4_Cl_2_]Cl are reported based on high quality single crystal X-ray diffraction data. The crystal structure of [Rh(NH_3_)_5_Cl]Cl_2_ was redetermined. All available crystal structures with octahedral complexes [RhCl*_x_*(NH_3_)_6-*x*_] were analyzed in terms of their packings and pseudo-translational sublattices. Pseudo-translation lattices suggest face-centered cubic and hexagonal closed-packed sub-cells, where Rh atoms occupy nearly ideal lattices.

## 1. Introduction

Rhodium(III) coordination compounds play an important role in Rh chemistry. Many Rh(III) species are suggested to be active homogeneous catalysts for practically important reactions. Rhodium organometallic chemistry has been investigated in details to support progress in chemical technology and catalysis [1,2]. Rhodium amines and acidocomplexes such as chlorides, nitrites, and sulfates play a fundamental role as intermediates in rhodium refinery from other platinum group metals (especially iridium). Rhodium(III) has an octahedral coordination, and as a result, substitution in its amine-coordinated compounds is kinetically difficult. Despite intensive research, not all theoretically possible rhodium(III) complexes have been isolated and structurally investigated in details, so far. Rhodium(III) coordinated with Cl and NH_3_ ligands can form 7 possible types of octahedral species [3]:

i) [RhCl_6_]^3–^;
ii) [Rh(NH_3_)Cl_5_]^2–^;
iii) *cis*– and *trans*–[Rh(NH_3_)_2_Cl_4_]^−^;
iv) *fac*– and *mer*–[Rh(NH_3_)_3_Cl_3_];
v) *cis*– and *trans*–[Rh(NH_3_)_4_Cl_2_]^+^;
vi) [Rh(NH_3_)_5_Cl]^2+^;
vii) [Rh(NH_3_)_6_]^3+^.

Among all possible compounds, salts with [RhCl_6_]^3–^ and [Rh(NH_3_)_5_Cl]^2+^ are the most common and have been investigated in great details. [Rh(NH_3_)_5_Cl]Cl_2_ seems to be the first investigated Rh(III) coordination compound [4] and was one of the first Rh(III) compound that was structurally characterized [5]. Crystal structures of fac–[Rh(NH_3_)_3_Cl_3_] and diverse salts with [Rh(NH_3_)_6_]^3+^ cations, as well as the first complexes with known anions [Rh(NH_3_)Cl_5_]^2–^ and *cis*–[Rh(NH_3_)_2_Cl_4_]^−^ were just recently reported [6,7,8]. No previous study have looked at the crystal structures of complexes with known *cis*–[Rh(NH_3_)_4_Cl_2_]^+^. Synthesis and properties of *trans*–[Rh(NH_3_)_2_Cl_4_]^−^ as well as mer–[Rh(NH_3_)_3_Cl_3_] have never been reported in the literature. As soon as the crystal structures of Rh(III) chloroamines were investigated during the past 80 years and briefly described in several sporadic publications, there was a need to perform a critical analysis of available experimental data and formulate general trends in their synthesis and crystal structures, especially to understand their packings in the context of stereochemistry.

Among the coordination compounds of transition metals with “simple” inorganic ligands, only crystal structures of [Pt(NH_3_)*_x_*Cl_1−*x*_] complexes containing Pt(IV) were systematically investigated and assessed from the crystallographic points of view [9]. At that time (70 years ago), all possible [Pt(NH_3_)*_x_*Cl_1−*x*_] complexes were synthesized, but not all crystal structures were known. Nevertheless, there were no efforts made to complete the Pt(IV) series or extend our knowledge about crystallography of coordination compounds of transition metals through a systematic investigation of other transition metals. Series of coordination compounds of other metals were investigated sporadically. In a later review [10], authors postulated the following. The main attention so far was paid to solve crystal structures of coordination compounds with organic ligands. At the same time, many crystal structures of coordination compounds with “simple” inorganic ligands were still unknown. Nevertheless, such complexes are important intermediates and target compounds that might be used as good models in understanding chemical reactions and processes for investigation of the chemical and structural aspects of coordination chemistry. The main idea of the current study was to fill this gap in experimental data regarding Rh(III) chloroamine complexes, obtain missing crystallographic information, and critically analyze existing experimental data on the synthetic procedures and crystal structures of Rh(III) chloroamines. The main goal was to understand the general trends in the Rh(III) crystal chemistry, as well as analyze the available crystal structures in terms of their packings and pseudo-translational sublattices. New crystal structures of (NH_4_)_2_[Rh(NH_3_)Cl_5_], *trans*–[Rh(NH_3_)_4_Cl_2_]Cl⋅H_2_O and *cis*–[Rh(NH_3_)_4_Cl_2_]Cl were solved and refined, and a crystal structure of the Claus salt, [Rh(NH_3_)_5_Cl]Cl_2_ was re-determined as well, based on high-quality single crystal X-ray diffraction data.

## 2. Review of the Available Synthetic Routines to Access Rh(III) Chloroamines

**Scheme 1:**
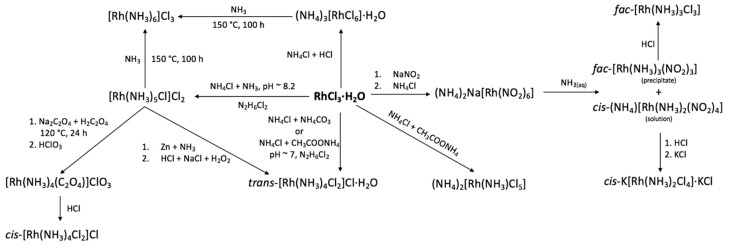
Synthetic pathways to access Rh(III) chloroamines.

RhCl_3_⋅*x*H_2_O is traditionally used as a starting chemical for the preparation of most Rh(III) complexes. All Rh(III) chloroamines could be in principle prepared by a reaction of RhCl_3_⋅*x*H_2_O with ammonia or ammonium chloride. Nevertheless, not all compounds are easily accessible due to the fast substitution of the first ligands in [RhCl_6_]^3−^ and [Rh(NH_3_)Cl_5_]^2−^ and difficulties in the substitution of the last Cl-ligand in [Rh(NH_3_)_5_Cl]^2+^. High interest of the [Rh(NH_3_)_5_Cl]^2+^ species indicates its practical importance for Rh(III) coordination chemistry. Schematically, synthesis of various rhodium(III) chloroamines are summarized in Scheme 1.

In general, ammonia substitution in Rh(III) is a relatively slow process and can be controlled by pH. pH control can be achieved using buffer solutions such as (NH_4_Cl + CH_3_COONH_4_) or (NH_4_Cl + NH_4_CO_3_), with a pH~7 and (NH_4_Cl + NH_3_) with a pH~8.2. (NH_4_)_2_[Rh(NH_3_)Cl_5_] first forms at neutral pH. In more basic media, [Rh(NH_3_)_3_Cl_3_] and [Rh(NH_3_)_4_Cl_2_]Cl as well as [Rh(NH_3_)_5_Cl]Cl_2_ can be isolated [11]. Due to the low trans-effect of coordinated ammonia, in comparison to Cl^−^ and NO_2_^−^, reaction of [RhCl_6_]^3−^ or [Rh(NO_2_)_6_]^3−^ with ammonia, results in a formation of *cis*–[Rh(NH_3_)_2_Cl_4_]^−^ and *cis*–[Rh(NH_3_)_2_(NO_2_)_4_]^−^, respectively [3]. Further nitration should result in a formation of fac–[Rh(NH_3_)_3_Cl_3_] and fac–[Rh(NH_3_)_3_(NO_2_)_3_], which was confirmed experimentally [6,7,8]. Due to the high stability of [Rh(NH_3_)_5_Cl]Cl_2_, its deamination cannot be performed to access mer–[Rh(NH_3_)_3_Cl_3_]. Only *trans*–[Rh(NH_3_)_2_Cl_4_]^−^ can be prepared from [Rh(NH_3_)_5_Cl]Cl_2_, using reactions with reducing agents (see synthesis 2.5 below). As far as we know, synthesis and properties of *trans*–[Rh(NH_3_)_2_Cl_4_]^−^ salts as well as mer–[Rh(NH_3_)_3_Cl_3_] were not described in the literature. Reaction with bidentate C_2_O_4_^2−^ was successfully applied for the substitution of amine- to acido-ligand: [Rh(NH_3_)_5_Cl]^2+^ + C_2_O_4_^2−^ → [Rh(NH_3_)_4_(C_2_O_4_)]^+^ + Cl^−^ + NH_3_ and further [Rh(NH_3_)_4_(C_2_O_4_)]^+^ + 2Cl^−^ → *cis*–[Rh(NH_3_)_4_Cl_2_]^+^ + C_2_O_4_^2−^. *trans*–[Rh(NH_3_)_4_Cl_2_]^+^ has been obtained by a direct reaction of [RhCl_6_]^3−^ with ammonia at high pH. It seems that, [Rh(NH_3_)_5_Cl]Cl_2_ is the most stable Rh(III) ammine complex and it can be prepared by deep amination of any rhodium compounds. The sixth chloride-ligand cannot be easily substituted and [Rh(NH_3_)_6_]Cl_3_ can only be prepared in an autoclave using concentrated water ammonia as a reaction medium. *cis*–[Rh(NH_3_)_4_Cl_2_]Cl has been synthetized by Hancock, using oxalate substitution [12] but its crystal structure was unknown.

Reducing agents such as ethanol, Zn-dust, and especially hydrazinium salts speed up a ligand’s substitution in Rh(III) complexes, due to a possible formation of highly reactive Rh(I) intermediates. Such catalytic reaction has been utilized to prepare *trans*–[Rh(NH_3_)_4_Cl_2_]Cl and [Rh(NH_3_)_5_Cl]Cl_2_ with high yields [11]. High stability of the Rh(III) complexes with nitrito-ligand can be used to access Rh(III) chloroamines, which can be can be obtained using a reaction of [Rh(NO_2_)_6_]^3−^ with ammonia and further with HCl [8,13,14]. In particular, such reaction can be utilized for preparation of fac–[Rh(NH_3_)_3_Cl_3_], *cis*–[Rh(NH_3_)_2_Cl_4_]^−^, and [Rh(NH_3_)Cl_5_]^2−^. Complicity of synthesis of *cis*–[Rh(NH_3_)_2_Cl_4_]^−^ salts can be explained by their relatively high solubility, in comparison with other Rh(III) chloroamines. Nevertheless, it seems that, *cis*–[Rh(NH_3_)_2_Cl_4_]^−^ has a high stability in solid-state and in solution. [Rh(NH_3_)_6_]Cl_3_ can be isolated from hydrothermal reaction in concentrated ammonia solutions.

Available synthetic protocols for known rhodium(III) chloroamines are briefly summarized below.

### 2.1. Ammonium Hexachlororhodate(III)

(NH_4_)_3_[RhCl_6_]⋅H_2_O can be isolated by slow evaporation of a 3 M HCl solution containing the stoichiometric amounts of RhCl_3_⋅*x*H_2_O and NH_4_Cl, with quantitative yield [15]; alternatively, according to [16,17], (NH_4_)_3_[RhCl_6_]⋅H_2_O can also be prepared by a direct reaction of H_3_[RhCl_6_] with NH_4_Cl water solution, or by evaporation of ammonium chloronitrorhodate(III) with HCl water solution. Water-free (NH_4_)_3_[RhCl_6_] is prepared by gentle heating of (NH_4_)_3_[RhCl_6_]⋅H_2_O in vacuum. For (NH_4_)_3_[RhCl_6_]⋅H_2_O, the IR-spectrum (cm^–1^) was: 3560, 3471 (broad) (ν(H_2_O)); 3174 (strong) (ν(NH_3_)); 1630 (broad) (δ_d_(H_2_O)); 1587 (δ_a_(NH_4_^+^)); 1381 (δ_s_(NH_4_^+^)); 325_ssh_, 318_s_, 314_ssh_ (ν(RhCl)); 205_m_, 196_sh_, 185_m_ (δ(ClRhCl)); 157_s_, 145_s_ (lattice) [15]; and the Raman-spectrum (cm^–1^) was: 339 (ν_1_(RhCl)); 300 (ν_2_(RhCl)); 283 (δ(ClRhCl)).

### 2.2. Ammonium Pentachloroaminerhodate(III)

(NH_4_)_2_[Rh(NH_3_)Cl_5_] can be prepared from Na_3_[RhCl_6_] or H_3_[RhCl_6_] through reaction with a mixture of ammonium chloride and ammonium acetate, in water [18]. In the current study, (NH_4_)_2_[Rh(NH_3_)Cl_5_] is prepared from (NH_4_)_3_[RhCl_6_]⋅H_2_O. 0.25 g of (NH_4_)_3_[RhCl_6_]⋅H_2_O and is dissolved in a minimum amount of hot water (several ml). A total of 2 mL of saturated water solution of NH_4_Cl is added. After heating for 5 min, 1.2 mL of ammonium acetate in water is added (ammonium acetate solution is prepared by neutralization of concentrated water ammonia with acetic acid). After heating for 1 h, dark-orange crystals are isolated. An additional portion is isolated by addition of 1 mL of ammonium acetate solution and evaporation until crystallization of orange needles. After cooling to room temperature, equal amounts of concentrated NH_4_Cl solution is added to the reaction mixture. Precipitate is filtered, washed with diluted NH_4_Cl water solution, and dried in air. Total yield was above 45%. Single crystals for the current single-crystal X-ray diffraction study were collected from the first portion. For (NH_4_)_2_[Rh(NH_3_)Cl_5_], the IR-spectrum (cm^–1^) was: 3370 (broad) (ν(NH_3_)); 3039 (shoulder) (ν_s_(NH_4_^+^)); 1680 (shoulder) (δ_d_(NH_4_^+^)); 1604, 1518 (δ_d_(NH_3_)); 1388 (δ_d_(NH_4_^+^)); 1279 (δ_s_(NH_3_)); and the Raman-spectrum (cm^–1^) was: 502 (ν(RhN)); 322, 305 (ν(RhCl)); 284, 218, 190, 130 (skeletal bending).

Crystals of K_2_[Rh(NH_3_)Cl_5_] can be prepared from K_2_[Rh(NH_3_)(NO_2_)_5_]∙H_2_O [8], by heating in 6 M HCl. After 24 h of crystallization at room temperature, dark-red crystals are filtered. For K_2_[Rh(NH_3_)Cl_5_], the IR-spectrum (cm^–1^) was: 3310, 3236 (ν(NH_3_)); 1604, 1519 (δ_a_(NH_3_)); 1292 (δ_s_(NH_3_)); 516 (ν(RhN)) [8].

### 2.3. Potassium cis–Tetrachlorodiamminerhodate(III)

According to [8,15], *cis*–K[Rh(NH_3_)_2_Cl_4_]⋅KCl can be prepared from (NH_4_)_2_Na[Rh(NO_2_)_6_]. As the first step, 5 g of RhCl_3_⋅*x*H_2_O is nitrated with 12 g of NaNO_2_ in 200 mL water. After heating and filtration, (NH_4_)_2_Na[Rh(NO_2_)_6_] is crystallized by addition of saturated NH_4_Cl water solution. Precipitate is filtered and added to ca. 5 mL of concentrated water solution of NH_3_, and boiled until its complete dissolution. The solution is cooled and filtrated from the fac–[Rh(NH_3_)_3_(NO_2_)_3_] precipitate. The residual clear solution is mixed with ca. 10 g of KCl and filtered immediately. After 3–4 days at room temperature, crystals of *cis*–K[Rh(NH_3_)_2_(NO_2_)_4_] are filtered. Both parts, crystals and solution, can be treated with HCl water solution (1:1). After short heating, yellow–orange solution are obtained; *cis*–K[Rh(NH_3_)_2_Cl_4_]⋅KCl can be crystallized by evaporation of the solution at room temperature [8]. For *cis*–K[Rh(NH_3_)_2_Cl_4_]⋅KCl, the IR-spectrum (cm^–1^) was: 3283 (strong), 3181 (weak) (ν(NH_3_)); 1604, 1548 (δ_a_(NH_3_)); 1287 (δ_s_(NH_3_)); 848, 793 (ρ_r_(NH_3_)); 506 (ν(RhN)) [8].

### 2.4. Fac–Trichlorotriaminerhodium(III)

According to [6,14], fac–[Rh(NH_3_)_3_Cl_3_] can be prepared by heating of fac–[Rh(NH_3_)_3_(NO_2_)_3_] with HCl (25 mL of 1:1 aqueous solution), for 3 h. After cooling the solution to room temperature, a light-yellow powder is isolated with a 91% yield. The crystalline structure of fac–[Rh(NH_3_)_3_Cl_3_] was solved and refined using powder X-ray diffraction data (PXRD). Fac–[Rh(NH_3_)_3_(NO_2_)_3_] can be prepared according to [19], by reaction of Na_3_[Rh(NO_2_)_6_] with sulfanilic acid and ammonia, or alternatively as a precipitate of heating of (NH_4_)_2_Na[Rh(NO_2_)_6_] with concentrated ammonia solution (see synthesis of *cis*–K[Rh(NH_3_)_2_Cl_4_]⋅KCl above). For fac–[Rh(NH_3_)_3_Cl_3_], the IR-spectrum (cm^–1^) was: 1590 (δ_a_(NH_3_)); 1318 (δ_s_(NH_3_)); 837 (ρ_r_(NH_3_)) [6].

### 2.5. Trans–Dichlorotetraminerhodium(III) Chloride

Synthesis of *trans*–[Rh(NH_3_)_4_Cl_2_]Cl was described by Lebedinskii [3] and later modified by other authors [11,20,21]. The method reported by Lebedinskii includes a reaction of RhCl_3_⋅*x*H_2_O with a water solution of NH_4_Cl and (NH_4_)_2_CO_3_. The mixture is heated on a steam bath for 3 h and cooled to room temperature for crystallization. After crystallization of a golden-yellow powder, the solid product is extracted by boiling with HCl (1:1 water solution). [Rh(NH_3_)_5_Cl]Cl_2_ is insoluble in HCl water solution but *trans*–[Rh(NH_3_)_4_Cl_2_]Cl can be easily dissolved and further recrystallized from diluted HNO_3_ water solution as *trans*–[Rh(NH_3_)_4_Cl_2_](NO_3_)⋅H_2_O [20].

Later, catalytic reaction with N_2_H_6_Cl_2_ was suggested to speed up ammonium substitution and improve the yield of *trans*–[Rh(NH_3_)_4_Cl_2_]Cl [9]. Briefly, (NH_4_)_3_[RhCl_6_]⋅H_2_O is dissolved in water after addition of NH_4_Cl powder, the mixture is boiled until a complete dissolution of the precipitate. Further addition of the CH_3_COONH_4_ water solution and a few milligrams of N_2_H_6_Cl_2_ powder, results in an immediate color change from red to golden-yellow. Additional portions of CH_3_COONH_4_ should be added to the reaction mixture to keep pH~7. After cooling to room temperature, a golden-yellow precipitate is filtered. For recrystallization, the *trans*–[Rh(NH_3_)_4_Cl_2_]Cl powder is dissolved in hot HCl (2:1) and evaporated until crystallization. *trans*–[Rh(NH_3_)_4_Cl_2_]Cl is dissolved in hot water and recrystallized by addition of HCl water solution. Yield is above 30%. It should be noted, that authors reported water-free salt [11]; nevertheless, later studies suggested that only *trans*–[Rh(NH_3_)_4_Cl_2_]Cl⋅H_2_O can be isolated. Such a catalytic reaction seems to be quite easy and reproducible to access *trans*–[Rh(NH_3_)_4_Cl_2_]Cl⋅H_2_O and its derivatives.

Alternatively, according to [21], *trans*–[Rh(NH_3_)_4_Cl_2_]Cl⋅H_2_O can by prepared from [Rh(NH_3_)_5_Cl]Cl_2_. A mixture of [Rh(NH_3_)_5_Cl]Cl_2_ and Zn dust is heated in a solution of 12 M ammonia and water (10:13) at 50 °C, for 5 min with stirring. The resultant mixture is filtered. The pale-yellow solution is cooled in an ice-bath. pH should be adjusted to ~6–7 by adding of 12 M HCl. NaCl, HCl, and H_2_O_2_ are sequentially added. The reaction mixture is rapidly boiled under reflux for 30 min; an orange solution is filtered. The precipitate is washed with 0.1 M HCl and the solution is kept in a refrigerator, overnight at 5 °C. The orange-yellow crystals are isolated by filtration and further recrystallized from 0.1 M HCl (50 °C), after addition of 6 M HCl and cooling; yield is above 70%.

For *trans*–[Rh(NH_3_)_4_Cl_2_]Cl⋅H_2_O, the IR-spectrum (cm^–1^) was: 3268, 3178, 3155 (ν(NH_3_)); 1604, 1542 (δ_a_(NH_3_)); 1298 (strong) (δ_s_(NH_3_)); 837, 820 (ρ_r_(NH_3_)); the Raman-spectrum (cm^–1^) was: 491, 506 (ν(RhN)); 306, 320(shoulder) (ν(RhCl)); 270, 259, 216, 205, 187, 158, 148 (skeletal bending).

### 2.6. Cis–Dichlorotetraminerhodium(III) Chloride

*Cis*–[Rh(NH_3_)_4_Cl_2_]Cl can be prepared from [Rh(NH_3_)_4_(C_2_O_4_)]ClO_4_⋅H_2_O by boiling it for 1 min, with 6 M HCl [13]. Bright-yellow solution is cooled in ice for crystallization of bright-yellow crystals. An additional portion can be obtained by addition of methanol. The product can be recrystallized from hot water by addition of 3 M HCl; yield is above 90%. [Rh(NH_3_)_4_(C_2_O_4_)]ClO_4_⋅H_2_O can be prepared from [Rh(NH_3_)_5_Cl]Cl_2_ [13]. A mixture of [Rh(NH_3_)_5_Cl]Cl_2_ (6.8 mmol), Na_2_C_2_O_4_ (6.8 mmol), and H_2_C_2_O_4_ (3.4 mmol) is dissolved in 70 mL of water, placed in a stainless steel autoclave, and heated at 120 °C for 24 h. Solution of [Rh(NH_3_)_4_(C_2_O_4_)]^+^ is filtered out and cooled to room temperature. Small volume of 70% HClO_4_ is added to the solution for a crystallization. Pale-yellow crystals are further purified from hot water by addition of water solution of LiClO_4_; yield is above 55%.

According to our experience, *cis*–[Rh(NH_3_)_4_Cl_2_]Cl can be prepared without intermediate crystallization of [Rh(NH_3_)_4_(C_2_O_4_)]ClO_4_⋅H_2_O. A mixture of [Rh(NH_3_)_5_Cl]Cl_2_ (6.8 mmol), Na_2_C_2_O_4_ (6.8 mmol), and H_2_C_2_O_4_ (3.4 mmol) is dissolved in 70 mL of water, placed in a stainless steel autoclave, and heated at 120 °C for 48 h. the reaction mixture should be heated with 1 mL of concentrated HCl water solution. After cooling to room temperature, light-yellow precipitate is filtered. Precipitate contains mainly unreacted [Rh(NH_3_)_5_Cl]Cl_2_. A light-orange solution is evaporated for several days on air at room temperature. Orange crystals are contaminated with minimum light-yellow [Rh(NH_3_)_5_Cl]Cl_2_ crystals, as well as with large light-yellow needles of an unknown crystal structure and composition. The procedure can be recommended for laboratory synthesis of *cis*–[Rh(NH_3_)_4_Cl_2_]Cl. Nevertheless, for preparation of large amounts of the salt, it should be further optimized.

For *cis*–[Rh(NH_3_)_4_Cl_2_]Cl, the IR-spectrum (cm^–1^) was: 3270, 3140 (ν(NH_3_)); 1600 (δ_a_(NH_3_)); 1300, 1290 (δ_s_(NH_3_)); 798, 818 (ρ_r_(NH_3_)). The Raman-spectrum (cm^–1^) was: 489, 506 (ν(RhN)); 305 (ν(RhCl)); 252, 217, 203 (skeletal bending).

### 2.7. Chloropentaminerhodium(III) Chloride (Claus Salt)

Similar to *trans*–[Rh(NH_3_)_4_Cl_2_]Cl⋅H_2_O, [Rh(NH_3_)_5_Cl]Cl_2_ can be prepared from RhCl_3_⋅*x*H_2_O, with high yield, using hydrazinium salts as catalysts [22,23]. In comparison with the synthesis of *trans*–[Rh(NH_3_)_4_Cl_2_]Cl⋅H_2_O, reaction should be performed in a more basic medium. Briefly, 1 g of RhCl_3_⋅*x*H_2_O powder is dissolved in 10 mL of concentrated HCl and heated for 0.5 h. After complete dissolution, 10–20 mg of solid N_2_H_6_Cl_2_ is added together with 20 mL of hot ammonia buffer, with pH~8.2. Immediately, the solution turns to light-yellow with a formation of [Rh(NH_3_)_5_Cl]Cl_2_ precipitate (precipitate might also contain minor quantities of Rh, [Rh(NH_3_)_3_Cl_3_] and *trans*–[Rh(NH_3_)_4_Cl_2_]Cl). The precipitate is filtered and washed from [Rh(NH_3_)_4_Cl_2_]Cl with hot HCl water solution (2:1). [Rh(NH_3_)_5_Cl]Cl_2_ can be purified by dissolution in hot water (insoluble Rh and [Rh(NH_3_)_3_Cl_3_] are left on the filter) and recrystallized by addition of an equal volume of 10 wt. % water solution of HCl. After 2–3 h, light yellow crystals of [Rh(NH_3_)_5_Cl]Cl_2_ are filtered and washed with ethanol and dried in air; yield is above 75 %. For [Rh(NH_3_)_5_Cl]Cl_2_, IR-spectrum (cm^–1^): 3288 (strong), 3173 (weak) (ν(NH_3_)); 1559 (δ_a_(NH_3_)) was; 1308(strong), 1273(weak) (δ_s_(NH_3_)); 850 (ρ_r_(NH_3_)); 492 (ν_a_(RhN)); 275, 266 (δ(NRhN)); 200 (ν(RhCl)); 102shs, 96s (lattice). The Raman-spectrum (cm^–1^) was: 506, 486 (ν(RhN)); 319 (ν(RhCl)); 266 (π(RhN)+ δ(NRhN)) [22,24].

### 2.8. Hexaminerhodium(III) Chloride

[Rh(NH_3_)_6_]Cl_3_ can be prepared from [Rh(NH_3_)_5_Cl]Cl_2_, according to [7,23]. [Rh(NH_3_)_5_Cl]Cl_2_ is heated with concentrated NH_3_ water solution in a 10-mL Teflon autoclave at 150 °C for 100 h. After natural cooling, the reaction mixture is washed with water and evaporated in air for several days. Dry colorless powder contains pure [Rh(NH_3_)_6_]Cl_3_. The procedure is quite general, any other rhodium(III) chloroamines, as well as rhodium(III) chloride can be used to prepare [Rh(NH_3_)_6_]Cl_3_ with nearly quantitative yield.

For [Rh(NH_3_)_6_]Cl_3_, the IR-spectrum (cm^–1^) was: 3276 (strong), 3181 (weak) (ν(NH_3_)); 1616, 1572, 1546 (δ_a_(NH_3_)); 1302 (strong), 1279 (weak) (δ_s_(NH_3_)); 855, 821, 755 (ρ_r_(NH_3_)); 470 (ν_a_(RhN)); 310 (δ_a_(NRhN)) [7]. The Raman-spectrum (cm^–1^) was: 515 (ν_s_(RhN)); 480 (ν_a_(RhN)); 240 (δ(NRhN)) [25].

Protocols 2.7 and 2.8 have been tested many times by several independent research groups and can be considered to be the easiest methods for a preparation of [Rh(NH_3_)_5_Cl]Cl_2_ and [Rh(NH_3_)_6_]Cl_3_ from various starting materials, including Rh(III) refinery as [Rh(NH_3_)_5_Cl]Cl_2_ from mixtures of transition metal chlorides. Both protocols give high yields of pure crystalline products.

## 3. Materials and Methods

For the current study, (NH_4_)_2_[Rh(NH_3_)Cl_5_] has been prepared according to [18], starting from (NH_4_)_3_[RhCl_6_]⋅H_2_O (see procedure 2.2 above). A total of 0.25 g (NH_4_)_3_[RhCl_6_]⋅H_2_O was dissolved in 5 mL hot water. A total of 2 mL of saturated water solution of NH_4_Cl was added. After heating for 5 min, 1.2 mL of ammonium acetate in water was added. After heating for 1 h and cooling to room temperature, dark-orange crystals were isolated.

Light-orange, plate-shaped, single crystals of *trans*–[Rh(NH_3_)_4_Cl_2_]Cl⋅H_2_O were prepared using catalytic reaction with hydrazinium chloride ([11], see procedure 2.5 above). A total of 0.64 g NH_4_Cl powder was added to 0.21 g (NH_4_)_3_[RhCl_6_]⋅H_2_O and dissolved in 10 mL water; mixture was boiled until a complete dissolution of the precipitate. Further addition of 0.27-g solid CH_3_COONH_4_ and a few milligrams of N_2_H_6_Cl_2_ powder, resulted in an immediate color change from red to golden-yellow. Additional portions of CH_3_COONH_4_ were added (max 0.1 g) to the reaction mixture to keep pH~7. After cooling to room temperature, a golden-yellow precipitate was filtered. Single crystals were collected from the stock solution, after its evaporation. A total of 1-mm light-yellow needles were collected from the surface of the solution, after completion of the reaction and cooling to room temperature (room temperature X-ray diffraction experiment). An additional portion of the crystals was obtained after evaporation of the solution after two weeks (crystals were used for low temperature X-ray diffraction experiment).

Light-orange, plate-shaped single crystals of *cis*–[Rh(NH_3_)_4_Cl_2_]Cl were prepared using an autoclave reaction of [Rh(NH_3_)_5_Cl]Cl_2_ with Na_2_C_2_O_4_ and H_2_C_2_O_4_, without isolation of the [Rh(NH_3_)_4_(C_2_O_4_)]ClO_4_⋅H_2_O intermediate ([13], see procedure 2.6 above). A mixture of 50 mg [Rh(NH_3_)_5_Cl]Cl_2_, 23 mg Na_2_C_2_O_4_, and 13 mg H_2_C_2_O_4_ was dissolved in 5 mL of water, placed in a stainless steel autoclave and heated at 120 °C, for 48 h. After finishing the first step, the reaction mixture was heated with 1 mL of concentrated HCl water solution. After cooling to room temperature, the light-yellow precipitate (unreacted [Rh(NH_3_)_5_Cl]Cl_2_) was filtered. Light-orange stock solution was evaporated for several days in air, at room temperature. Orange crystals of *cis*–[Rh(NH_3_)_4_Cl_2_]Cl were contaminated with minimum light-yellow [Rh(NH_3_)_5_Cl]Cl_2_ crystals, as well as with large light-yellow needles of an unknown crystal structure and composition.

[Rh(NH_3_)_5_Cl]Cl_2_ was prepared using a catalytic procedure with hydrazinium chloride ([7,23], see procedure 2.7 above). A total of 1 g of RhCl_3_⋅*x*H_2_O powder was dissolved in 10 mL of concentrated HCl and heated for 0.5 h. After a complete dissolution, a few milligrams of solid N_2_H_6_Cl_2_ was added together with 20 mL of hot ammonia buffer, with pH~8.2. Immediately, the solution turned to light-yellow, with a formation of [Rh(NH_3_)_5_Cl]Cl_2_ precipitate. The precipitate was filtered and washed from [Rh(NH_3_)_4_Cl_2_]Cl, with hot HCl water solution (2:1). A total of 10 mg of [Rh(NH_3_)_5_Cl]Cl_2_ powder was dissolved in hot water and evaporated at room temperature to get [Rh(NH_3_)_5_Cl]Cl_2_ single crystals.

Fourier transform infrared spectroscopy of (NH_4_)_3_[RhCl_6_]⋅H_2_O, (NH_4_)_2_[Rh(NH_3_)Cl_5_], and *trans*–[Rh(NH_3_)_4_Cl_2_]Cl⋅H_2_O (FTIR, Digi-lab Excalibur FTS 3000 FTIR) was recorded in transmission in the range of 4000–500 cm^-1^, using the ART optic (MK11 Golden Gate system). Raman spectra were collected on a Raman ART optic spectrometer MK11 Golden Gate system.

The single crystal X-ray diffraction (SC-XRD) study of *trans*–[Rh(NH_3_)_4_Cl_2_]Cl⋅H_2_O and [Rh(NH_3_)_5_Cl]Cl_2_ single crystals was performed on an automated Bruker APEX-II CCD diffractometer (MoKα radiation, graphite monochromator, and two-dimensional CCD detector) at 150 K at the BAM. The structure was refined in the anisotropic approximation. Hydrogen atoms were set geometrically. All calculations were performed using the SHELXTL software [26]. For the refinement of *trans*–[Rh(NH_3_)_4_Cl_2_]Cl⋅H_2_O at 150 K, in the final full-matrix refinement of 88 structural parameters, the total number of reflexes used was 3813 and the divergence factors were: *R*_all_ = 2.78%, *wR*_ref_ = 8.49%; for 3747 reflections with *I* ≥ 2σ(*I*), *R*_gt_ = 2.75%, *wR*_gt_ = 8.48%, *S* factor against *F*^2^ was 1.179. For the refinement of *cis*–[Rh(NH_3_)_4_Cl_2_]Cl at 150 K, in the final full-matrix refinement of 43 structural parameters, the total number of reflexes used was 1186 and the divergence factors were: *R*_all_ = 4.19%, *wR*_ref_ = 12.27%; for 1185 reflections with *I* ≥ 2σ(*I*), *R*_gt_ = 4.19%, *wR*_gt_ = 12.27%, *S* factor against *F*^2^ was 1.359. For the refinement of [Rh(NH_3_)_5_Cl]Cl_2_ at 150 K, in the final full-matrix refinement of 49 structural parameters, the total number of reflexes used was 2347 and the divergence factors were: *R*_all_ = 5.99 %, *wR*_ref_ = 13.35 %; for 2099 reflections with *I* ≥ 2σ(*I*), *R*_gt_ = 5.11 %, *wR*_gt_ = 12.85 %, *S* factor against *F*^2^ was 1.085.

The X-ray diffraction study of *trans*–[Rh(NH_3_)_4_Cl_2_]Cl⋅H_2_O and (NH_4_)_2_[Rh(NH_3_)Cl_5_] single crystals was performed on an automated Bruker APEX-II CCD diffractometer (MoKα radiation, graphite monochromator, and two-dimensional CCD detector), at room temperature, at the Nikolaev Institute. For the refinement of *trans*–[Rh(NH_3_)_4_Cl_2_]Cl⋅H_2_O at room temperature, in the final full-matrix refinement of 94 structural parameters, the total number of reflexes used was 3928 and the divergence factors were: *R*_all_ = 4.64%, *wR*_ref_ = 10.56%; for 3308 reflections with *I* ≥ 2σ(*I*), *R*_gt_ = 3.79%, *wR*_gt_ = 10.04%, *S* factor against *F*^2^ was 1.038. For the refinement of (NH_4_)_2_[Rh(NH_3_)Cl_5_] at room temperature, in the final full-matrix refinement of 66 structural parameters, the total number of reflexes used was 1886 and the divergence factors were: *R*_all_ = 1.93%, *wR*_ref_ = 4.29%; for 1748 reflections with *I* ≥ 2σ(*I*), *R*_gt_ = 1.71%, *wR*_gt_ = 4.20%, *S* factor against *F*^2^ was 1.078.

## 4. Crystal Structures of Rh(III) Chloroamines

Crystallographic characteristics of Rh(III) chloroamines are summarized in Table 1.

**Figure 1:**
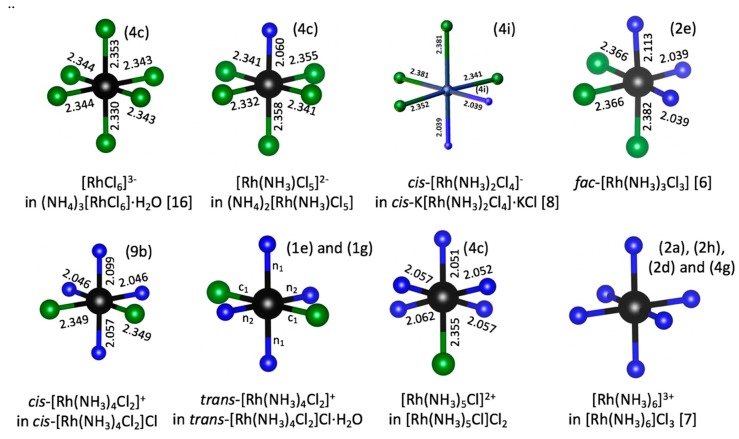
Coordination polyhedrons of Rh(III) (black) and their point symmetry in [RhCl*_x_*(NH_3_)_6−*x*_] crystal structures with ammonium or chloride as counter ions (see Table 1 for crystallographic details). Green balls represent Cl-ligand, blue—NH_3_. For *trans*–[Rh(NH_3_)_4_Cl_2_]Cl⋅H_2_O, n_1_ = 2.062 (1e) and 2.055 (1g), n_2_ = 2.060 (1e) and 2.053 (1g), c_1_ = 2.332 (1e) and 2.328 (1g) Å; interatomic distances for four crystallographically independent RhN_6_ cations, in crystal structures of [Rh(NH_3_)_6_]Cl_3_ are given in the text.

**Figure 2:**
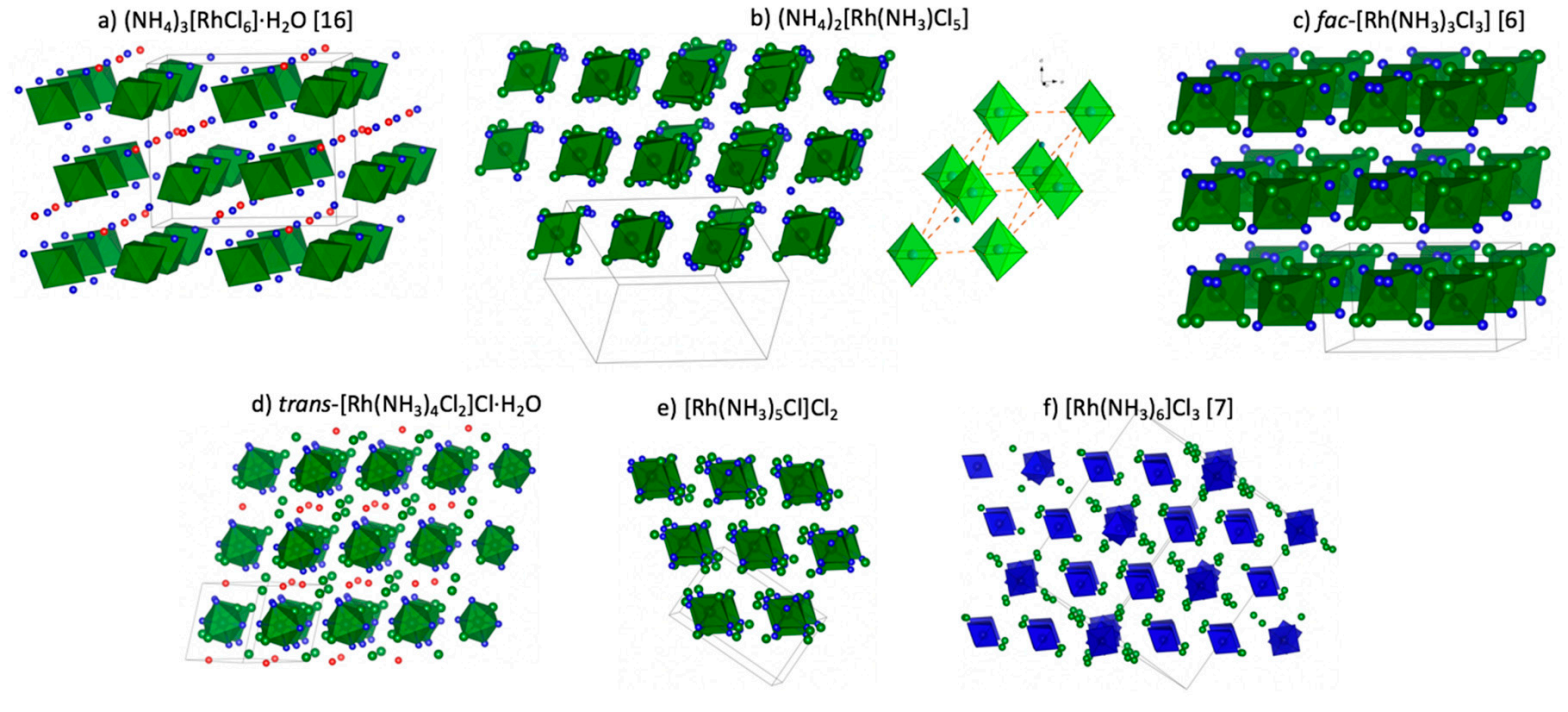
Crystal structures of [RhCl*_x_*(NH_3_)_6-*x*_] species: (**a**) (NH_4_)_3_[RhCl_6_]⋅H_2_O along *b*-direction, two layered hexagonal closed-packed layers are formed perpendicular to the *a*-direction; (**b**) three layered face-centered closed packing in (NH_4_)_2_[Rh(NH_3_)Cl_5_], corresponding pseudo-translational sub-lattice is shown; (**c**) crystal structure of fac–[Rh(NH_3_)_3_Cl_3_] along the *c*-direction, hexagonal layers can be found perpendicular to the *a*-direction; three layered face-centered closed packing in crystal structure of *trans*–[Rh(NH_3_)_4_Cl_2_]Cl⋅H_2_O (**d**), [Rh(NH_3_)_5_Cl]Cl_2_ (**e**), and [Rh(NH_3_)_6_]Cl_3_ (**f**) (Rh atoms are shown as black circles, N atoms are blue, Cl atoms are green, and H atoms are omitted for clarity).

Coordination of Rh(III) in all crystal structures are shown in Figure 1. Crystal structures of all coordination compounds mentioned above are summarized in Figure 2. Almost all salts had a relatively low crystal symmetry and crystallized in triclinic, monoclinic, and especially in orthorhombic (*Pnma*) space groups. Only *cis*–[Rh(NH_3_)_4_Cl_2_]Cl crystallized in the *R*3*m* space group. It should be noted that in all cases the highest symmetry of Rh(III) octahedron is *m*, even in potentially symmetric fac–[Rh(NH_3_)_3_Cl_3_], [RhCl_6_]^3−^, and [Rh(NH_3_)_6_]^3+^. Nevertheless, in all described complexes, Rh(III) had a nearly ideal octahedral coordination. Deviations of valence angles from 90° between Cl and NH_3_ ligands were relatively small and usually below 5°. All crystals structures had relatively dense packing, without formation of any channels or cages. As a result, almost all complexes crystallized without crystal water. Only (NH_4_)_3_[RhCl_6_]⋅H_2_O had crystal water in its structure. In all existing examples, such as (NH_4_)_2_[Rh(NH_3_)Cl_5_] and K_2_[Rh(NH_3_)Cl_5_] as well as (NH_4_)_3_[RhCl_6_]⋅H_2_O and K_3_[RhCl_6_]⋅H_2_O, ammonium and potassium salts seemed to be isomorphous to each other, but the sodium salts usually showed different crystal structures [15,28]. It should be noted that previously reported water-free *trans*–[Rh(NH_3_)_4_Cl_2_]Cl, according to [21] and the current study seemed to have crystal water and should be written as *trans*–[Rh(NH_3_)_4_Cl_2_]Cl⋅H_2_O.

In the crystal structure of (NH_4_)_3_[RhCl_6_]⋅H_2_O, [RhCl_6_]^3–^ anions occupy special positions corresponding to mirror planes, where Rh and two Cl ligands sit on the plane. The crystal structure is quite regular with only N…Cl short contacts of about 3.21–3.28 Å.

Crystal structures of (NH_4_)_2_[Rh(NH_3_)Cl_5_] and K_2_[Rh(NH_3_)Cl_5_] [8] are very similar and belong to a large series of isostructural isoformular A_2_[ML_5_X] complexes. In their crystal structures, [Rh(NH_3_)Cl_5_]^2–^ cations lie on the mirror plane. Rh, NH_3_, trans-Cl, and two cis-Cl ligands occupy positions in the mirror plane; two cis-Cl ligands are symmetrically connected by the plane. Rh...Rh distances are 6.364–7.274 Å. Isostructural similarity of (NH_4_)_2_[Rh(NH_3_)Cl_5_] and [Rh(NH_3_)_5_Cl]Cl_2_ salts is the main important structural feature within the series. If in [Rh(NH_3_)_5_Cl]Cl_2_ all Cl positions were filled with nitrogen ligands and all nitrogen positions were filled with Cl atoms, nearly ideal crystals structures of (NH_4_)_2_[Rh(NH_3_)Cl_5_] can be obtained. Both crystal structures had similar cell parameters. Such a phenomenon has been also found for isomorphous (NH_4_)_2_[Ir(NH_3_)Cl_5_] and [Ir(NH_3_)_5_Cl]Cl_2_ salts, as well as (NH_4_)_2_[V(NH_3_)Cl_5_] and [Rh(NH_3_)_5_Cl]Cl_2_ complexes [29,31]. In both types of crystal structures, the Rh(III) octahedra occupy positions on the mirror plane. Both structures have deformed CaF_2_ fluorite structure types, where coordination ions occupy Ca positions and contra-ions correspond to the F positions. (NH_4_)_2_[Rh(NH_3_)Cl_5_] and [Rh(NH_3_)_5_Cl]Cl_2_ can be considered as anti-isomorphic pairs of crystal structures. As soon as both (NH_4_)_2_[Rh(NH_3_)Cl_5_] and [Rh(NH_3_)_5_Cl]Cl_2_ have similar crystal structures, it would be interesting to prepare their co-salts to check a possibility to get isomorphous double salts, without miscibility such as (NH_4_)_2–2*x*_[Rh(NH_3_)Cl_5_]_1–*x*_[Rh(NH_3_)_5_Cl]*_x_*Cl_2+2x_, double salts with a fixed composition with NH_4_Cl in its structure, similar to *cis*–K[Rh(NH_3_)_2_Cl_4_]⋅KCl, such as [Rh(NH_3_)Cl_5_][Rh(NH_3_)_5_Cl]⋅2NH_4_Cl, or double complex salt such as [Rh(NH_3_)Cl_5_][Rh(NH_3_)_5_Cl], similar to [Rh(NH_3_)Cl_5_][IrCl_6_] [32,33]. It would be also interesting to investigate temperature-induced ligand exchange in such double salts, in the solid-state.

Anion *cis*–[Rh(NH_3_)_2_Cl_4_]^−^ has been crystallized only as a double salt with KCl, namely *cis*–K[Rh(NH_3_)_2_Cl_4_]⋅KCl [8]. The Rh central atoms occupied a mirror plane as deformed octahedrons [RhN_2_Cl_4_]. The valence angles divided from 90° less than 3.7°. The shortest Rh...Rh distances in the structure were ca. 5.727 Å. K^+^ cations occupied two different positions—inversion center with six Cl^−^ neighbors and mirror planes with seven Cl^−^ neighbors. The shortest Rh…K distances were 3.835 and 3.914 Å.

Crystal structure of fac–[Rh(NH_3_)_3_Cl_3_] has been solved and refined using powder X-ray diffraction data [6]. The monoclinical crystal structure was solved using the direct space approach and was refined using the Rietveld method. In its crystal structure, Rh(1), Cl(1), and N(1) atoms were arranged on a mirror plane. Rh—Cl and Rh—N bond lengths that lay on the mirror plane, were 0.016 (4) and 0.074 (6) Å longer than the others. Deviations of the valence of cis angles on the Rh(1) atom from the ideal value of 90° did not exceed 6.8°. Isolated fac–[Rh(NH_3_)_3_Cl_3_] octahedrons formed nearly ideal flat hexagonal layers. Layers were oriented perpendicular to the *c* axis, with the distances between the layers being equal to *c*. All layers followed a simple arrangement without shirts (*AA* packing type).

The crystal structure of *trans*–[Rh(NH_3_)_4_Cl_2_]Cl was obtained based on poor experimental data [21]. Positions of water molecules were not found and refined. So, details of the crystal structure of *trans*–[Rh(NH_3_)_4_Cl_2_]Cl⋅H_2_O were described here for the first time. The crystal structure contained two independent *trans*–[Rh(NH_3_)_4_Cl_2_]^+^ cations; both cations were located in inversion centers. The shortest Cl^−^…N distances were 3.44–3.48 Å, Cl…N were 3.28 Å, and the crystal water showed O…N (3.26 and 3.45 Å) and O…Cl (3.38 Å) contacts.

**Figure 3:**
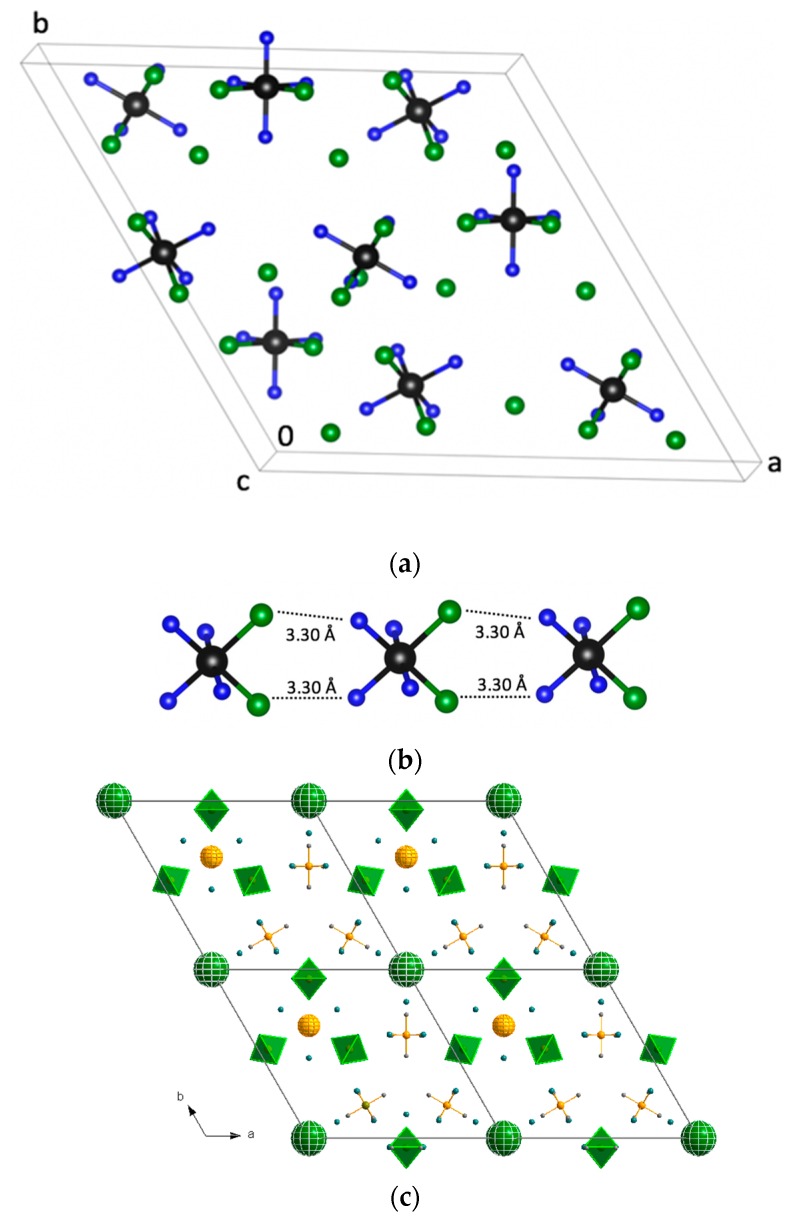
Crystal structure of *cis*–[Rh(NH_3_)_4_Cl_2_]Cl along the *c*-direction (Rh atoms are shown as black circles, nitrogen atoms are blue, chlorine atoms are green, and the hydrogen atoms are omitted for clarity) (**a**); right: chain along the *c*-direction with the shortest Cl…N contacts (**b**). Three-layered packing in the crystal structure of *cis*–[Rh(NH_3_)_4_Cl_2_]Cl. First layer of *cis*–[Rh(NH_3_)_4_Cl_2_]^+^ is shown as polyhedrons, the second layer is shown in ball-and-stick stile. *cis*–[Rh(NH_3_)_4_Cl_2_]^+^ cations have hexagonal arrangement with empty space, which is shown as large balls (the green balls belong to the first layer, yellow balls belong to the second layer, and the small balls represent Cl^−^ anions) (**c**).

*Cis*–[Rh(NH_3_)_4_Cl_2_]Cl was synthetized in the early 1960s but its crystal structure was unknown. The crystal structure of the salt had a high symmetry with only one crystallographically independent *cis*–[Rh(NH_3_)_4_Cl_2_]^+^ cation located on the mirror plane. Rh and two N ligands sat on the mirror plane. Cl^−^….N contacts were 3.32–3.36 Å. The cations formed chains parallel to the *c*-direction with relatively short Cl…N contacts of 3.30 Å (Figure 3). The crystal structure had three-layered packing of *cis*–[Rh(NH_3_)_4_Cl_2_]^+^ cations with empty space. Cl^−^ anions occupied hexagonal voids.

In the crystal structure of [Rh(NH_3_)_6_]Cl_3_ [7], there were four non-equal [Rh(NH_3_)_6_]^3+^ cations with Rh–N distances of 2.043–2.072 Å. The N–Rh–N angles were close to 90°. There were close contacts between the coordinated NH_3_ ligands and the Cl^−^ anions, of about N…Cl 3.27–3.50 Å.

All crystal structures had a relatively high packing density and could be described as a closed packing of coordination cations or anions with contra-ions placed in the octahedral or tetrahedral voids of such packings. To find the general orientation of the closed packed layers, a pseudo-translational sublattice could be applied. Pseudo-translation sublattices can be applied to find a minimum sublattice with highest symmetry. Pseudo-translation ordering of structural fragments that can be corresponded to heavy atoms or groups of atoms, such as polyhedral for coordination compounds with the presence of smaller sublattices with higher symmetry, in comparison to the actual symmetry of the unit cell. To find these pseudo-translation sublattices, at least three intersecting crystallographic planes should be found. Crystallographic independent planes should correspond to the heaviest structural fragments, where their concentration is high [34,35]. The suitable planes can be found from a powder diffraction pattern as diffraction reflexes with the strongest intensity. Therefore, the general accurate motif of the crystal structure can be described as the most symmetric pseudo-translation sublattice. Briefly, the procedure for selecting a pseudo-translation sublattice should include a choice of the triad of diffraction lines with the strongest diffraction intensity. The diffraction indices of these reflexes should be for a matrix with a determinant (*D*) equal to the number of heavy fragments in the unit cell (*N*_hf_). Corresponding intersections between such plains (*h*_1_ *k*_1_ *l*_1_), (*h*_2_ *k*_2_ *l*_2_), and (*h*_3_ *k*_3_ *l*_3_) form a sublattice with a number of heavy fragments equal to *D*. The corresponding vectors (***a***_T_, ***b***_T_ and ***c***_T_) should give the nearest sites of the initial lattice and angles (α_T_, β_T_ and γ_T_) between the most symmetric arrangement of the crystal structure. Their length *a*_T_, *b*_T_, *c*_T_, should correspond to the minimal distances between the heavy fragments in the crystal structure (Table 1). Detailed mathematics behind the technique can be found in [34,35,36]. Finally, the shortest sublattice should be found with the corresponding angles close to 60°, 90°, 109.5°, and 120°, which is usually a sign for the most symmetric sub-cells. Orientation of pseudo-translational sub-lattices should correspond to the layers with the highest packing density in the crystal structure.

Among all crystal structures, only (NH_4_)_3_[RhCl_6_]⋅H_2_O corresponds to the hexagonal closed packed lattice of [RhCl_6_]^3–^ anions, with hexagonal layers arranged with *AB* stacking perpendicular to the *a*-direction in the crystal structure. Distances between the layers correspond to *a*_T_ = 7.01 Å (Figure 2). Two ammonia cations occupy tetrahedral voids in the packing. Crystal water and the third ammonia cation corresponds to the octahedral positions of the packing. Water-free (NH_4_)_3_[RhCl_6_] seems to be isostructural to (NH_4_)_3_[IrCl_6_] [30]. In the crystal structure of (NH_4_)_3_[IrCl_6_], translational sublattice correspond to ideal pseudo-rhombohedral sub-cells with angles close to α_T_ ≈ 60.3° and three nearly equal axis of 7.32 Å. Such an arrangement correspond to the face-centered cubic packings of three hexagonal layers with *ABC* stackings. Ammonia occupy all tetragonal and all hexagonal voids in the structure.

**Figure 4:**
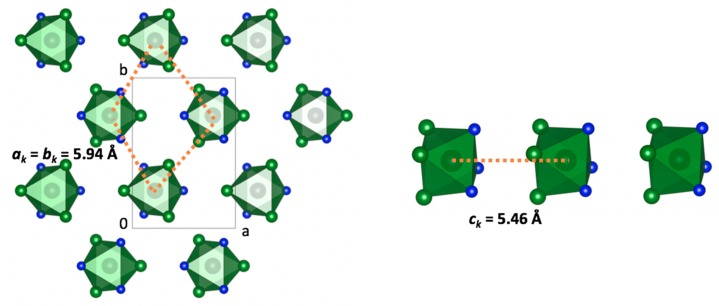
Left: crystal structure of fac–[Rh(NH_3_)_3_Cl_3_] [4] along the *c*-direction (Rh atoms are shown as black circles, nitrogen atoms are blue, chlorine atoms are green, and the hydrogen atoms are omitted for clarity); right: stacking of fac–[Rh(NH_3_)_3_Cl_3_] octahedrons parallel to the *c*-direction. Dotted lines show pseudo-trigonal sub-cell.

In the crystal structure of fac–[Rh(NH_3_)_3_Cl_3_], the hexagonal layers of isolated fac–[Rh(NH_3_)_3_Cl_3_] octahedrons was observed, perpendicular to the *c*-direction (Figure 4). The shortest distances between the octahedrons in the layers corresponded to *a*_T_ = *b*_T_ = 5.94 Å. Hexagonal layers formed quite unusual *AA* stacking along the *c*-direction, which could be driven by the interactions between the triangle {….[(NH_3_)_3_RhCl_3_]…[(NH_3_)_3_RhCl_3_]…} faces (Figure 4, left).

In all other structures, the metrics obtained corresponded to the pseudo-rhombohedral sub-cells with angles close to α_T_ ≈ 60–70° and two nearly equal longer axis (6.90–7.30 Å) and one shorter axis (6.40–7.10 Å). Such an arrangement corresponded to the face-centered cubic packings of three hexagonal layers with *ABC* stackings. As an example, the correspondence of closed-packed layers and pseudo-rhombohedral sub-cell for (NH_4_)_2_[Rh(NH_3_)Cl_5_] is shown in Figure 2. In the crystal structures of (NH_4_)_2_[Rh(NH_3_)Cl_5_], *trans*–[Rh(NH_3_)_4_Cl_2_]Cl⋅H_2_O, [Rh(NH_3_)_5_Cl]Cl_2_, and [Rh(NH_3_)_6_]Cl_3_, the hexagonal layers corresponded to the packings of the coordination ions, ammonia in (NH_4_)_2_[Rh(NH_3_)Cl_5_] could be found in the tetrahedral voids; coordinated ammonia was placed in the octahedral voids of the closed-packed structure. Cl^−^ in the crystal structure of [Rh(NH_3_)_5_Cl]Cl_2_ was located in the tetrahedral voids and coordinated Cl was placed in the octahedral voids of the packing. Similar, Cl^−^ anions in *trans*–[Rh(NH_3_)_4_Cl_2_]Cl⋅H_2_O could be found in the tetrahedral voids, crystal water was located in the octahedral positions of the packing. In [Rh(NH_3_)_6_]Cl_3_, two Cl^−^ anions were located in the tetragonal voids, one Cl^−^ anion corresponded to the octahedral void in the *ABC* packing of [Rh(NH_3_)_6_]^3+^ cations.

The crystal structure of *cis*–[Rh(NH_3_)_4_Cl_2_]Cl showed a relatively low packing density, where the hexagonal layers had empty space. Cl^−^ anions occupied hexagonal voids in the structure. N…Cl interactions between the *cis*–[Rh(NH_3_)_4_Cl_2_]^+^ cations should be mentioned. The structure had a hexagonal sublattice with angles α_T_ = β_T_ = 60.1°, γ_T_ = 120°. The shortest pseudo-translational vector, *c*_T_ = 6.87 Å, corresponded to the distances between the pseudo-hexagonal layers.

## 5. Conclusions

Rh(III) is known as a metal with rich coordination chemistry in solution and in solid-state. In the last decade, nearly all possible octahedral Rh(III) chloroamine complexes were synthesized and structurally characterized. Some rare compounds were prepared just recently. Synthetic procedures available in the Rh(III) chemistry included direct substitution of Cl^−^ to NH_3_ in solution, as well as catalytic reactions. In some cases, NO_2_^−^- and C_2_O_4_^2−^-coordinated derivatives could be used as intermediates. Such approaches were never successful in Ir(III) chemistry, where only the direct substitution can be applied. For synthesis of Pt(IV), oxidation of the Pt(II) species with Cl_2_ in the solution is typical. Such routes are impossible in Rh(III) chemistry.

Synthetic and crystal chemistry of Cr(III), Co(III), Pt(IV), Ir(III), and Ir(IV) compounds have been investigated in many details; nevertheless, only [Rh(NH_3_)*_x_*Cl_1-*x*_] complexes containing Rh(III) give a nearly complete series available for detailed systematic analysis of their transformations and details of their crystal chemistry. For iridium, the existence of Ir(III) and Ir(IV) complexes with identical coordination spheres was experimentally confirmed. [IrCl_6_]^3−^ and [IrCl_6_]^2−^, as well as pairs of [Ir(NH_3_)Cl_5_]^2−^/[Ir(NH_3_)Cl_5_]^−^ and [Ir(NH_3_)_4_Cl_2_]^2+^/[Ir(NH_3_)_4_Cl_2_]^+^ with Ir(III) and Ir(IV), respectively, were available for investigation [31]. Among all Rh(III) [Rh(NH_3_)*_x_*Cl_1−*x*_] complexes only mer–[Rh(NH_3_)_3_Cl_3_] was not described in the literature; further efforts to synthetize and characterize such coordination compounds should be made.

[Rh(NH_3_)*_x_*Cl_1−*x*_] complexes did not show a large variety in their crystal structures. All crystal structures could be described to have a closed-packing coordination of ions, with the contra-ions and crystal water located in octahedral or tetrahedral voids. (NH_4_)_3_[RhCl_6_]⋅H_2_O showed hexagonal closed-packed structure, fac–[Rh(NH_3_)_3_Cl_3_] showed a relatively rare, one-layer packing due to the formation of specific contacts between non charged fac–[Rh(NH_3_)_3_Cl_3_] octahedrons. Such one-layer packing might be stable only under curtain *P*–*T* conditions and small changes in pressure or temperature, as well as crystallization from other solvents might be revealed in the formation of other polymorphic modifications of fac–[Rh(NH_3_)_3_Cl_3_], similar to several known polymorphic modifications isolated for fac–[Rh(NH_3_)_3_(NO_2_)_3_].

The crystal structures of (NH_4_)_2_[Rh(NH_3_)Cl_5_], *trans*–[Rh(NH_3_)_4_Cl_2_]Cl⋅H_2_O, [Rh(NH_3_)_5_Cl]Cl_2_, and [Rh(NH_3_)_6_]Cl_3_ had face-centered cubic arrangement of closed, packed hexagonal layers. Such an arrangement could be easily found, based on pseudo-translational sublattices. In all mentioned salts, pseudo-translational sublattices had similar metrics. Directions of pseudo-translational vectors gave the orientation of the closed-packed layers in their crystal structures. It is important to note that the crystal structures of (NH_4_)_2_[Rh(NH_3_)Cl_5_] and [Rh(NH_3_)_5_Cl]Cl_2_ were similar. It is possible that this is a general tendency in coordination chemistry, which should be proven for other classes of isoformular coordination compounds. Such a phenomenon might be useful as a tool for crystal structure prediction and preparation of double complexes.

The Rh(III) chloroamine complexes did not realize the maximal possible symmetry of coordination polyhedrons. Deformations of the coordination polyhedrons depended not only on the ligand arrangements, but also possibly on the packings of deformed octahedrons in the crystal structures. Closed-packings are typical for such structurally rigid coordination species, but local structure and symmetry of coordination polyhedrons usually fit the packings of hexagonal packed layers. Changes in the interatomic distances were also high due to trans-effect in cis- and trans-isomers. If specific interactions could be realized in the crystal structures (similar to fac–[Rh(NH_3_)_3_Cl_3_]) due to sterically-driven factors, then close-packed arrangements could possibly distort with a formation of other types of crystal packings.

## Figures and Tables

**Table 1:** Crystallographic data for [RhCl*_x_*(NH_3_)_6−*x*_] complexes.

Composition	(NH_4_)_3_[RhCl_6_]⋅H_2_O^a^ SC-XRD, RT	(NH_4_)_2_[Rh(NH_3_)Cl_5_] SC-XRD, RT	K_2_[Rh(NH_3_)Cl_5_] SC-XRD, RT	*cis–*K[Rh(NH_3_)_2_Cl_4_]⋅KCl SC-XRD, RT	*fac*–[Rh(NH_3_)_3_Cl_3_] PXRD, RT	*trans–*[Rh(NH_3_)_4_Cl_2_]Cl SC-XRD, RT	*trans–*[Rh(NH_3_)_4_Cl_2_]Cl⋅H_2_O SC-XRD, RT	*trans–*[Rh(NH_3_)_4_Cl_2_]Cl⋅H_2_O SC-XRD, 150 K	*cis–*[Rh(NH_3_)_4_Cl_2_]Cl SC-XRD, 150 K	[Rh(NH_3_)_5_Cl]Cl_2_ SC-XRD, 150 K	[Rh(NH_3_)_6_]Cl_3_ SC-XRD, 150 K
[Ref.]	[16,17]	[current study]	[8]	[8]	[6]	[27]	[current study]	[current study]	[current study]	[current study]^b^	[6]
Colour	ruby–red	orange–red	dark–red	red	light–yellow	pale–yellow	pale–yellow	light–orange	yellow	light–yellow	colourless
Space group	*Pnma* (62)	*Pnma* (62)	*Pnma* (62)	*C*2/*m* (12)	*P*21*/m* (11)	*P*$\bar{1}$ (2)	*P*$\bar{1}$ (2)	P$\bar{1}$ (2)	*R*3*m* (160)	*Pnma* (62)	*C*2*/m* (12)
*a*, Å	12.213(3); 12.193(4)	13.3982(3)	13.464(6)	11.822(5)	6.72793(26)	6.35(2)	5.8697(7)	5.8341(6)	19.600(4)	13.2770(13)	12.6244(10)
*b*, Å	7.012(1); 7.007(4)	10.2926(2)	9.989(4)	11.608(5)	9.78879(38)	5.77(2)	6.4606(7)	6.4469(7)	19.600(4)	10.4930(9)	21.499(2)
*c*, Å	14.151(3); 14.167(10)	6.8177(2)	6.932(3)	8.196(4)	5.46106(20)	13.81(5)	12.5870(13)	12.5189(12)	6.3357(13)	6.6578(6)	12.9439(16)
α, °	90	90	90	90	90	115.3(2)	85.412(5)	94.430(4)	90	90	90
β, °	90	90	90	107.56(2)	90	99.6(2)	89.721(6)	90.029(4)	90	90	113.188
γ, °	90	90	90	90	90	77.5(2)	77.767(5)	102.407(4)	120	90	90
*Z*	4	4	4	4	2	2	2	2	9	4	12
*V,* Å^3^	1211.9(5); 1210.38(2)	940.18(4)	932.23(7)	1072.42(8)	358.2	445.0(5)	464.95(9)	458.42(8)	2107.9(9)	927.54(15)	3229.33
ICSD	61129; 202826	1965928	430397	430395	426435	—	1965934	1965942	1965940	1965941	187073
*h*_1_ *k*_1_ *l*_1_	0 1 1	0 1 1	0 −1 1	−1 1 1	1 1 0	0 0 −2	0 1 0	0 1 0	2 0 2	0 1 1	−1 −3 1
*h*_2_ *k*_2_ *l*_2_	–1 –2 0	0 1 −1	0 –1 –1	–1 –1 1	1 −1 0	1 0 0	1 0 1	1 0 1	0 2 2	0 1 −1	−1 3 1
*h*_3_ *k*_3_ *l*_3_	2 0 0	−2 −1 0	2 1 0	−2 1 0	0 0 1	0 1 0	−1 0 1	−1 0 1	0 0 3	−2 −1 0	0 0 −2
***a*_T_**	***– a***	***−a/*4 *+ b*/2 *+ c*/2**	***a*/4 − *b*/2 − *c*/2**	***b*/2 + *c*/2**	***a*/2 + *b/*2**	***c*/2**	***b***	***b***	***a/2***	***−a*/4 *+ b*/2 *+ c*/2**	**−*a*/2 − *b*/6**
***b*_T_**	**−*b*/2−*c*/2**	***− a/*4 *+ b*/2 *− c*/2**	***a/*4 *− b*/2 *+ c*/2**	***−b*/2 *+ c*/2**	***a*/2 + *b*/2**	***a***	***a*/2 + *c*/2**	***a*/2 *+ c*/2**	***b/2***	***a/*4 *− b*/2 *+ c*/2**	**−*a*/2 + *b*/6**
***c*_T_**	***a*/2 *− b*/4 *− c*/4**	***−a*/2**	***a*/2**	***−a*/2 *− c*/2**	***c***	***b***	**−*a*/2 + *c*/2**	**−*a*/2 *+ c*/2**	***a*/3 *+ b*/3 - *c*/3**	***−a*/2**	**−*a*/2 − *c*/2**
*a*_T_, Å	7.01	7.02	6.95	7.11	5.94	6.91	6.46	6.44	9.80	7.04	7.26
*b*_T_*,* Å	7.90	7.02	6.95	7.11	5.94	6.35	6.96	6.90	9.80	7.04	7.26
*c*_T_. Å	7.27	6.70	6.73	6.09	5.46	5.77	6.93	6.90	6.87	6.64	7.04
$\bar{a_{T},b_{T}c_{T}}$*^,^* Å	7.39	6.91	6.88	6.77	5.78	6.34	6.78	6.75	-	6.91	7.19
α_T_, °	57,1	61.5	61.03	102.7	92.9	77.5	50.0	49.9	61.6	61.9	59.2
β_T_, °	76,1	61.5	61.03	102.7	92.9	64.7	91.0	91.1	61.6	61.9	62.3
γ_T_, °	63,6	58.1	59.84	109.6	111.0	80.4	80.7	80.8	120	56.4	59.2
$\bar{\alpha_{T},\beta_{T}\gamma_{T}}$^,^ °	65.6	60.4	60.6	105.0	98.9	74.2	73.9	73.9	-	60.1	60.2

Bold correspond to vectoctor, all letters in the line represent corresponding translational vectors. a) no experimental data for (NH_4_)_3_[RhCl_6_], it is probably isostructural to (NH_4_)_3_[IrCl_6_] [28]: *P*$\bar{1}$ (2), *a* = 12.602(30), *b* = 7.226(20), *c* = 7.502(20) Å, α = 119.18(59), β = 91.60(50)°, γ = 106.70(50)°. For (NH_4_)_3_[IrCl_6_], *a*_T_ = (−2 0 0) = *−**a***/2 *− **c***/2 = 7.24, *b*_T_ =(0 1 0) = *b* = 7.23, *c*_T_ = (1 0 −1) = *−**c*** = 7.50 Å, α_T_ = 60.8, β_T_ = 60.4, γ_T_ = 59.8°***;*** b) previous data for [Rh(NH_3_)_5_Cl]Cl_2_ (SC-XRD, RT [29,30]): *Pnma* (62), *a* = 13.36(1), *b* = 10.46(1), *c* = 6.741(8) Å, *V* = 942.0 Å^3^, ICSD 1524334.
